# Self-Assembling Peptides and Their Application in the Treatment of Diseases

**DOI:** 10.3390/ijms20235850

**Published:** 2019-11-21

**Authors:** Sungeun Lee, Trang H.T. Trinh, Miryeong Yoo, Junwu Shin, Hakmin Lee, Jaehyeon Kim, Euimin Hwang, Yong-beom Lim, Chongsuk Ryou

**Affiliations:** 1 Department of Pharmacy and Institute of Pharmaceutical Science and Technology, Hanyang University, Gyeonggi-do 15588, Korea; guranye@hanyang.ac.kr (S.L.); sho_ymr0623@naver.com (M.Y.); dugalle1@naver.com (J.S.); gkrals92@naver.com (H.L.); rlawoguses@naver.com (J.K.); 2 Department of Materials Science and Engineering, Yonsei University, Seoul 03722, Korea; euimin92@naver.com (E.H.); yblim@yonsei.ac.kr (Y.-b.L.)

**Keywords:** peptide, self-assembly, nanostructure, drug delivery, disease

## Abstract

Self-assembling peptides are biomedical materials with unique structures that are formed in response to various environmental conditions. Governed by their physicochemical characteristics, the peptides can form a variety of structures with greater reactivity than conventional non-biological materials. The structural divergence of self-assembling peptides allows for various functional possibilities; when assembled, they can be used as scaffolds for cell and tissue regeneration, and vehicles for drug delivery, conferring controlled release, stability, and targeting, and avoiding side effects of drugs. These peptides can also be used as drugs themselves. In this review, we describe the basic structure and characteristics of self-assembling peptides and the various factors that affect the formation of peptide-based structures. We also summarize the applications of self-assembling peptides in the treatment of various diseases, including cancer. Furthermore, the in-cell self-assembly of peptides, termed reverse self-assembly, is discussed as a novel paradigm for self-assembling peptide-based nanovehicles and nanomedicines.

## 1. Introduction

The development of effective drug delivery systems and patient-customized therapies has recently emerged as a popular research topic. The ability to control the production of functional materials at the nanometer level is currently being explored for various medical applications. Nanomedicines, in the forms of nanospheres, nanoparticles, and other nanostructures modified with antibodies, peptides, glycans, and carbon, offer an alternative approach to classical drugs through their potential selectivity for diseased cells. The Food and Drug Administration (FDA) has approved abraxane, a nanomedicine for metastatic breast cancer, which encapsulates the anticancer drug paclitaxel within protein (albumin) nanoparticles [1,2]. Other anticancer drugs such as doxorubicin [3], 5-fluorouracil [4], 10-hydroxycamptothecin [5], and methotrexate [6] were also used to fabricate nanomedicines with albumin. Alternatively, gelatin was used to fabricate protein-based nanomedicines to increase drug loading efficiency and extend the duration of drug release [7]; unlike albumin, which can only encapsulate hydrophilic compounds, gelatin can be used to encapsulate both hydrophobic and hydrophilic drugs. Despite the success in the controlled release of drugs from protein-conjugated nanostructures, cellular targeting and cellular delivery of drugs have remained challenging.

Based on the success of conjugation of drugs with biocompatible proteins, researchers developed peptide-based nanomedicines using small peptides designed for the control of drug release and targeting. The small peptides are biocompatible and biodegradable. Furthermore, these peptides can be easily modified, thus inducing various self-assembled structures with different shapes, depending on the biochemical environment. The lower occurrence of side effects and stable drug release are also advantages of peptide-based self-assembled structures [2,8]. Peptide self-assembly is a process in which peptides spontaneously form ordered aggregates [9]. Hydrogen bonding, hydrophobic interactions, electrostatic interactions, and van der Waals forces combine to maintain the peptide-based self-assembled structures in a stable low-energy state [8]. In addition to the building blocks of self-assembling peptides, the research has also focused on self-assembled nanostructures with different shapes [10], including micelles, vesicles, and fibrillar structures such as nanotubes and fibers [11,12,13,14]. Based on the characteristics of the self-assembling peptides, the self-assembled structures can be used for intracellular or targeted tissue delivery of various nucleotides and antibodies for therapy, and for the delivery of drugs that cannot be easily mobilized owing to their physicochemical characteristics or those that exhibit a rapid clearance rate. In addition, nanostructures composed of self-assembling peptides can be applied to the treatment of various diseases as peptide drugs.

In this review, we have described the types of self-assembling peptides and their associated characteristics, and have discussed the principles of peptide self-assembly. Furthermore, we have examined the applications of self-assembling peptides in disease treatment. Finally, the in-cell self-assembly of peptides, termed “reverse engineering of peptide self-assembly,” is explored as a new approach to deliver peptide-based nanostructure to cells.

## 2. Self-Assembling Peptides: Structure and Characteristics

Self-assembling peptides comprise monomers of short amino acid sequences or repeated amino acid sequences that assemble to form nanostructures. Peptide assemblies show distinctive physicochemical and biochemical activities, depending on their morphology, size, and accessibility of the reactive surface area. In most cases, morphological control is the initial step in the design of functional peptide assemblies. For amphiphile molecules, the concept of the molecular packing parameter offers a simple and intuitive insight into morphological control. The molecular packing parameter, P, is calculated as $P=V_{0}/al_{0}$, where $V_{0}$ is the volume, $l_{0}$ is the length of the hydrophobic tail, and a is the surface area per molecule [15]. The relationship between P and the shape of molecular assemblies is as follows: P<13 for spherical micelles, 13<P<12 for cylindrical micelles, 12<P<1 for flexible bilayers or vesicles, P≈1 for planar bilayers, and P>1 for inverted micelles. In short, the morphology transitions from more highly curved assemblies to less curved structures as the packing parameter increases. This feature can also be found in amyloid fibrils, which have been linked to various diseases in their natural state and produce more stable and functional structures through various amino acid combinations. The building blocks described below can be used in the design of nanostructures by considering the molecular and chemical properties of amino acids and peptides.

### 2.1. Building Blocks

The building blocks of self-assembled peptide structures can be categorized by their different constituent amino acids and the various bound chains or motifs. The characteristics of some peptide building blocks are summarized in Table 1.

#### 2.1.1. Dipeptides

Dipeptides are the simplest building block in peptide nanotechnology. The diphenylalanine peptide (L-Phe-L-Phe; FF) is the core recognition motif of the Alzheimer’s *β*-amyloid peptide [16]. Many studies have indicated that the peptide and its derivatives can self-assemble into highly ordered structures and other forms with nanoscale order [16,17,40]. These building blocks are used in the production of functional peptide nanotubes for casting molds of metal nanowires or electrochemical biosensing platforms [41,42,43]. Aromatic interactions are suggested to play a key role in the tubular structures. Other tubular structures are also produced by N-terminal modification of diphenylalanine to a non-charged FF analog, such as Boc–F–F–COOH, Z-F–F–COOH and Fmoc–F–F–COOH (Boc: tert-butoxycarbonyl; F: phenylalanine; Z: N-Carbobenzoxy; Fmoc: 9-fluorenylmethoxycarbonyl) [44]. β-Amino acids, which provide notable structural diversity through their extra C–C bond, are also used in dipeptide self-assembly. The derivatives of β-amino acids form hydrogels by self-assembly and exhibit prolonged bioavailability relative to α-amino acid derivatives [18,45].

#### 2.1.2. Surfactant-Like Peptides

Surfactant-like peptides are characterized by their large reductive effect on the surface tension of water and their solubility in both organic solvents and water. Their solubility stems from the amphiphilic structure of the peptide, with several consecutive hydrophobic residues that constitute the hydrophobic tail, and one or two hydrophilic charged residues that serve as the head [20]. Often, surfactant-like peptides include a hydrophilic head group of negatively charged aspartic acid at the C-terminus, thus containing two negative charges, and a lipophilic tail made of hydrophobic amino acids such as alanine (A), valine (V), or leucine (L); the acetylated N-terminus has no charge. When dissolved in water, these surfactant-like peptides tend to self-assemble to shield the hydrophobic tail from contact with water. As with lipids and fatty acids, the supramolecular structure is characterized by the formation of a polar interface that sequesters the hydrophobic tail from water. Aspartic acid (D) and glutamic acid (E) have hydrophilic characteristics with a negative charge. Lysine (K), histidine (H), and arginine (R) also have hydrophilic characteristics, but they are positively charged. In contrast, glycine (G), alanine (A), valine (V), leucine (L), and isoleucine (I) are hydrophobic. By directional organization, Ac-AAAAAAD (A6D), Ac-VVVVVVD(V6D), and positively charged Ac-AAAAAAK (A6K), or any other design, can be used for surfactant-like peptide design. Zhao [20], Wang et al. [22], and Vauthey et al. [21] suggest that, through self-assembly, nanotubes or nanovesicles are the main structures formed by surfactant-like peptide assembly, and that they can function in a manner similar to lipid detergent micelles on the lipid bilayer of cells.

#### 2.1.3. Peptide Amphiphiles with an Alkyl Group

Most self-assembling peptides have very simple structures: A hydrophobic tail with a hydrophilic head. In this group of peptides, the link with the hydrophobic alkyl chains is the most common modification in the peptide building blocks. When the alkyl chain combined with a peptide block is exposed to aquatic solutions, the hydrophobic tail of the peptide adopts a three-dimensional (3D) structure, similar to protein folding. Usually, the peptides form nanofibers, micelles, vesicles, nanotapes, or nantotubes. Hargerink et al. [23] developed a mineralized self-assembling peptide, including an alkyl tail and phosphorylated serine residues, to interact with calcium. A C16 alkyl tail with a VVVAAAEEE (V3A3E3) peptide was reported to form a gel under pressure or through electrostatic interaction with divalent cations, and this gel functioned as a scaffold for mesenchymal stem cell or three-dimensional culture [23,46].

#### 2.1.4. Bolaamphiphilic Peptides

The difference between surfactant-like peptides and bolaamphiphiles is the number of hydrophilic heads of the building block. The surfactant-like peptide building block has only one hydrophilic head, whereas the bolaamphiphile has two hydrophilic heads connected by a hydrophobic section [47]. The double-headed design results in special properties and a highly complex assembly phenomenon in bolaamphiphilic molecules. Notably, bolaamphiphilic molecules can possess different head groups at either end of the hydrophobic chain; these are called asymmetric bolas [48]. For example, one end of the bolaamphiphilic molecule can be functionalized with amine groups to bind negatively charged nucleotides, and it can assemble to form vesicles through amphiphilic properties [48]. Bolaamphiphilic peptides are related to amyloid-like aggregation. For example, K and R in KAAAAK (KA4K), KAAAAAAK (KA6K), and RAAAAAAR (RA6R) bolaamphiphilic peptides have hydrophilic character and are connected by the hydrophobic A residues. Their assembled product has a fibrous form [26]. Another bolaamphiphilic peptide, EFLLLLFE (EFL4FE), which contains an E residue, shows a flat membrane extension and forms peptide nanotubes by concentration differences [28]. As the charge of amino acids is altered in different pH conditions, based on their molecular properties, these bolaamphiphilic peptides may aggregate or disaggregate according to the environmental pH [26]. Bolaamphiphiles are a category of emerging nanomaterials with the ability to self-assemble into various valuable nanostructures [49,50,51].

#### 2.1.5. Ionic-Complementary Self-Assembling Peptides

The study of ionic-complementary peptides began from research on the Z-DNA binding protein, which includes the unusual 16-amino acid sequence of AEAEAKAKAEAEAKAK (EAK16). This peptide shows a unique pattern of charge distribution and forms membrane-like structures [52]. The ionic-complementary peptides are characterized by an alternating arrangement of negatively and positively charged residues. According to their charge distribution, these peptides can be classified to three types: Type I, +- block; Type II, ++-- block; Type III, +++---; and Type IV, ++++---- block; in these, the charged amino acid repeats work like “molecular Lego” to assemble the structure [31]. To design other peptide blocks, additional ionic-complementary peptides can be combined and modified. The charge distribution is a major force determining the peptide structure; for example, --++--++ shows α-helical periodicity and -+-+ has β-strand periodicity. RADA16 is another ionic-complementary self-assembling peptide. RADA 16-I (RADARADARADARADA) has the charge distribution pattern of +-+-+-+-, whereas RADA16-II (RARADADARARADADA) has the charge distribution pattern of ++--++--, but both form β-sheets after assembly. Many alternative compositions of ionic complementary self-assembling peptides show α, β, or random coil structures after assembly, but transition between α and β has also been reported [53]

#### 2.1.6. Cyclic Peptides

Cyclic peptides are easily explained by the stacking of amino acids to form a cylindrical structure. There is an intermolecular hydrogen bond between each amino acid, forming a β-sheet-like tubular structure. By stacking, the amino acid side chains are located outside the cylinder and the peptide backbone is located on the inner side of the cylinder [54]. The external surface properties and the internal diameter can be controlled by the appropriate choice of amino acid side chains and the number of amino acids employed in the cyclic peptide [35,55]. Cyclic peptides have advantages over linear peptides, owing to their stable conformations and the conformational stability of the exposed surface [37]. A recent study from Jeong et al. [38] suggested the use of a hybrid cyclic peptide for a more stable α-helical structure. From the results of α-helical stabilization with carbon nanotubes, the covalent linker peptide connected to the side chains decreased the conformational entropy of the unfolded state, resulting in α-helix stabilization between the target molecule and self-assembling peptide.

### 2.2. Formation of Nanostructures

Self-assembled peptide nanostructures are formed by the designed building blocks. The nanostructure of self-assembling peptides can be classified into several types based on their constructed results, such as fibers, cylinders, or flat forms. Micelles are also classified as self-assembled nanostructures. These differences arise from the hydrophobic interaction of peptides in aqueous solutions and are dependent on the building block designs. Here, we have summarized the classified nanostructures of self-assembling peptides.

#### 2.2.1. Nanofibers

The self-assembling peptide EAK16 sequences with periodically repeating positive and negative charges form a stable structure by ionic-complementary forces in a checkerboard-like pattern and then assembles typical β-sheet structures, eventually forming a hydrogel network of nanofibers [52]. Generally, nanofibers have a diameter of less than 100 nm. Aqueous solutions, including ions with different pH, are generally used to produce nanofibers from self-assembling peptide building blocks. In recent research, a light-induced self-assembling peptide was developed by modification of the amino acid sequences, and nanofibers were produced [56]. Peptide amphiphiles with an alkyl group are the most renowned self-assembling peptides that form nanofibers. The designed peptides may include a specific sequence for RGD binding, fluorescence, or any other small molecule in their tails [57,58]

#### 2.2.2. Nanotubes

The structure of nanotubes is similar to that of the nanofibers mentioned above. However, they are elongated nanostructures with a hole on the inner side of the capillary. Recent research has focused on the development of non-covalent nanotubes, owing to their advantages in self-organization and easy control of the nanotube diameters. The most commonly used materials for nanotubes are cyclic peptides. Cyclic peptide nanotubes are formed by the stacking of peptides with high stability compared with other peptide building blocks. Drugs can be loaded inside these tubes and can be conjugated or bound to the outside of tubes; therefore, nanotube-based peptide assemblies have a wide range of applications in drug delivery [59]. The cyclic peptide, cyclo[-(L-Gln-D-Ala- L -Glu- D -Ala)_2_-] with an even number of alternating D- and L-amino acids, forms a distinct structure of nanotubes [35]. Amphiphilic and surfactant-like peptides also form nanotubes with lipidic or surfactant characteristics by self-assembly [21,60]. For example, the peptide diphenylalanine, which is the core recognition motif of Alzheimer’s β-amyloid polypeptide, with an uncharged peptide can successfully produce nanotubes [44]. NH_2_–F–F–COOH is efficiently self-assembled into a tubular structure that was most likely to have an antiparallel β-sheet conformation, but acetylation to form Ac–F–F–COOH resulted in a structure that did not dissolve in either water or fluoroalcohols. These results indicate that non-charged peptide blocks were better for nanotube synthesis.

#### 2.2.3. Nanoparticles

Nanoparticles are diverse and are formed by different building blocks. The structures range from nanospheres with a hollow core to various solid structures [61]. The charged amphiphilic block co-polypeptides [poly(L-lysine)-b-poly(L-leucine)] self-assemble to form stable vesicles and micelles in aqueous solutions [62]. Their hydrophobicity contributes to their rigidity and stability. Moreover, the guanidine residue of arginine increases the cell-penetrating actions to facilitate the delivery of encapsulated materials such as drugs. The temperature-responsive self-assembling peptide, elastin-like polypeptide (ELP), is a linear di-block peptide, but in response to a temperature change, it forms spherical micelles upon drug loading. The sensitivity of ELP to temperature can be controlled by increasing the number of ELP units. Cyclic peptides also produce vesicle-forming nanostructures. Shirazi et al. reported that the [WR]4 peptide successfully functioned as a drug delivery vehicle with molecular cargo, with a circular vesicle-like structure ranging from 25 to 60 nm in size [63].

#### 2.2.4. Nanotapes

β-Alanine-histidine dipeptide and lysine–threonine–threonine–lysine–serine pentapeptide each conjugated to C16 palmitoyl hydrophobic lipid chains (C16-βAH and C16-KTTKS) form the stacks of β-sheets structures, resulting in nanotapes [64]. C16-βAH self-assemble into fibrils due to the hydrophobicity of the lipid tail. Self-assembly of C16-KTTKS [65] is controlled by pH or temperature; if the pH decreases to 4, the morphological transition from tape to fibrils occurs, but if the pH decreases further to 3, the nanotaper structure reforms. Lipopeptides of bacterial origin also form nanotapes, as described by Hamley et al. [66]. *Bacillus subtilis* produces a lipopeptide comprising a cyclic peptide head with different alkyl chains. This bio-originated molecule forms either micelles or nanotapes. The nanotape structures of self-assembling peptides often interact with each other and form double-layers. If the concentration of these nanotapes exceeds a certain threshold, they tend to form hydrogels

#### 2.2.5. Hydrogels

A hydrogel is a polymer network that is cross-linked or entangled. The properties of hydrogels formed from self-assembling peptides depend on pH, ionic strength, and temperature [67]. Some hydrogels can absorb large amounts of water and they can be designed to possess distinct structural elements with adjustable mechanical properties, similar to natural tissues. Peptide-based hydrogels are highly biocompatible, biodegradable, and simple [68,69,70]. The simplest dipeptide building block modified with Fmoc, diphenylalanine (Fmoc-FF), was found to form hydrogels comprised of nanofibril networks in aqueous solutions [71]. Modification of these peptides to Fmoc-FRGD and Fmoc-RGDF showed that the inclusion of the RGD motif also produced a hydrogel structure, but that it was not stable above pH 6.5. Hydrogel formation is not limited to a simple block structure. Alkyl chain peptide blocks form β-sheets by hydrophobic collapse and can also form aqueous gels [23]. Stable β-sheet structures produced by self-assembling peptides form hydrogels when the peptide block concentration is increased.

## 3. Factors for Peptide Self-Assembly

### 3.1. pH

pH is an important factor in determining the peptide structure. pH fluctuation results in changes in hydrogen bonds and salt bridges, which influence peptide structure [72]. A change in pH affects the charge of the side chains through protonation and deprotonation. These changes in amino acids result in disruption of the hydrogen bonds among amino acid residues and broken salt bridges, the ionic bonds formed between the positively and the negatively charged side chains of amino acids.

The peptide -ETATKAELLAKYEATHK- motif includes negatively charged amino acids towards the N-terminus and positively charged amino acids towards the C-terminus, conferring an α-helical structure. At pH 4, this peptide exhibits the clearest α-helical structure. However, the α-helical structure of the peptide turns to a structure similar to β-sheet at pH 8 [73]. The secondary structure of this peptide can be changed by pH fluctuation. Another self-assembling peptide, cyclic α, α-disubstituted α-amino acid (dAA), is a cyclic acetal that changes to acyclic dAA at low pH. The peptide, including the dAA side chain, is stabilized as an α-helix structure. However, structural changes in dAA induced by low pH affects the changes in the secondary structure of the peptide from an α-helix to a random coil [74]. The hydrophobic peptide -YVIFL- also demonstrates pH-dependent structural changes. The peptide forms an amorphous aggregate at pH 2. Protonation of -YVIFL- below pH 2 reduces the electrostatic and hydrogen bonds among peptides. This results in the formation of aggregates with an antiparallel stacking structure. At pH 9 and 11, electrostatic and hydrogen bonds are replaced, while the aggregates still maintain an antiparallel stacking structure [75]. Similarly, the glutamic acid of the -FKFEFKFEFKFE- peptide becomes hydrophobic by protonation at low pH. Thus, this peptide can aggregate through hydrophobic interaction and the aggregated structure can be maintained in the inner space of endosomes or lysosomes. When fused to the oligo-arginine R_12_ peptides, this hydrophobic peptide segment recruits itself and induces the formation of nanovesicle structures at low pH, which demonstrate stronger anti-prion activity and lower cytotoxicity than oligo-arginine R_12_ without the -FKFEFKFEFKFE- peptide [76]. These examples suggest the potential of using pH-dependently altered peptides to optimize peptide targeting inside the cells.

### 3.2. Temperature

Peptides that self-assemble by temperature variation initially exist as monomers, but when heated, they change forms to nanofibrils, micelles, and other peptide structures, such as non-specific aggregate-like networks, depending on the calcium concentration and pH of buffer [77]. Examples of such temperature-dependent self-assembling peptides include FF peptide [78], ELP (repeating VPGXG) [79], and the lauryl-VAGERGD peptide. FF forms a characteristic crystal nanowire structure during the process of heating to 90 °C and cooling to 25 °C. Heating FF to 90 °C reduces the ionization constant of -NH_3_^+^, making it highly soluble. However, if the temperature is lowered, the ionization constant increases and enhances the hydrogen bonds. As a result, self-assembly occurs, forming a nanowire structure [80]. ELP exists in a monomer form at temperatures below the transition temperature, but monomers are changed to micelles by heat energy. The increased heat energy to ELP micelles results in aggregates that form gels. By heating the ELP molecule, hydrophobic activity is increased, which results in the formation of a micellar structure in a polar solvent [78]. The backbone of the lauryl-VVVAGERGD peptide is stable at approximately 300 to 358 K and disintegrates at higher temperatures. However, rapid heating to high temperatures can also reshape the peptide backbone. This enables the system to bypass energy barriers and reach more thermodynamically stable configurations [81]. This process results in the peptide backbone, forming a thermodynamically stable nanofiber structure without disintegration [82].

**Figure 1:**
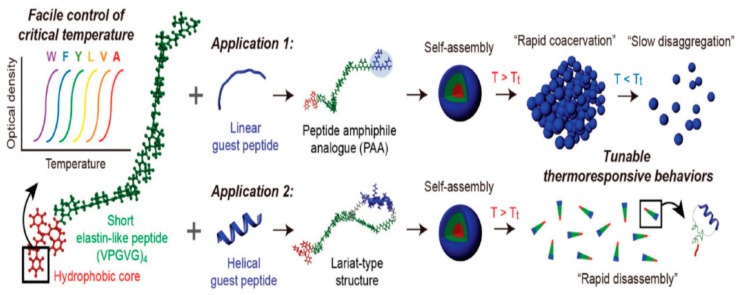
Peptide platform based on an miniaturized elastin-like peptide (MELP) for developing elastin-like peptide (ELP) amphiphiles with a thermo-responsive behavior that can be controlled by varying the types of conjugated guest–peptides, macromolecular topologies, and N-terminal amino acid residues. Reprinted with permission from [83].

Lim and coworkers reported that guest-associated ELP peptides with different additional nonpolar amino acid appendages showed disparate assembly behavior (Figure 1). They demonstrated the changes in thermo-responsive phase transition between miniaturized elastin-like peptides (MELPs) with different numbers of phenylalanine residues or different nonpolar amino acid composition. After establishing the tunable temperature-responsive system with short peptides, they investigated whether these peptides could be used as a platform for the development of thermo-responsive ELP amphiphiles with various functionalities. They coupled the MELP platform to a short linear RGD peptide and grafted an α-helical guest peptide to the platform in a lariat-type structure. The peptide platform devised by this group provides a new insight into the development of stimuli-responsive materials with wide ranging applications, including temperature-responsive drug delivery and controlled modulation of protein–protein interactions [83].

### 3.3. Other Stimuli

Redox activity is another factor that mediates self-assembly of peptides. Phenylalanine derivatives conjugated to naphthalene diimide (NDI) form a nanofibril structure in high ionic strength aqueous solvents. This nanofibril changes to a non-fibril aggregate in a reductive environment; however, the nanofibrillar structure can reform in re-oxidation conditions [84]. Electrolytes are one of the factors that induce self-assembly in peptides. The peptide Ac–(AEAEAKAK)_2_–CONH_2_ is found in the yeast protein zuotin. This peptide has a β-sheet structure composed of hydrophilic and hydrophobic residues and exists in a monomer form. When an electrolyte is added, electrostatic repulsion between the peptides is reduced and hydrophobic interactions and hydrogen bonding increase, causing self-assembly and in-parallel alignment of the β-sheet structures [85].

**Figure 2:**
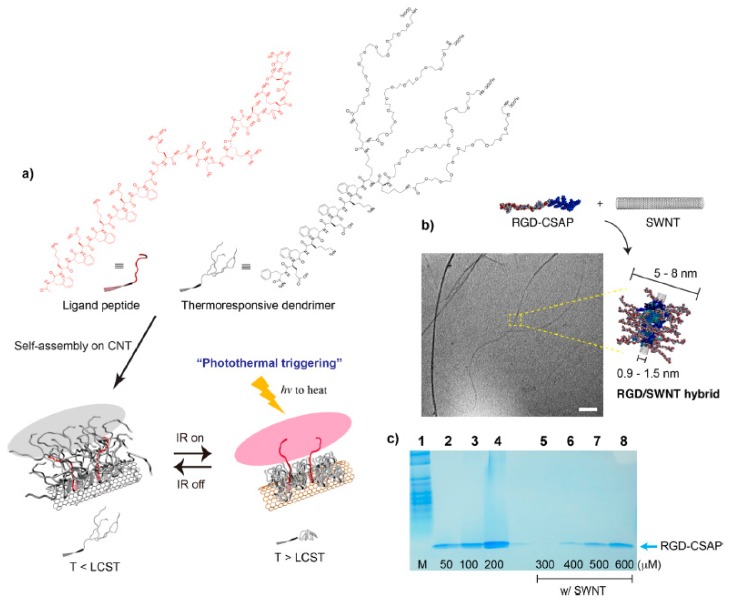
Photothermal control of multivalent ligand presentation. (**a**) Self-assembly of the ligand RGD peptide and thermoresponsive dendrimers on a carbon nanotube (CNT), followed by photothermal triggering of multivalent RGD ligands. (**b**) Transmission electron micrograph of an RGD/CNT hybrid. (**c**) SDS-PAGE analysis of the RGD complex that binds to CNT. Reprinted with permission from [86].

Light can also be a factor regulating the behavior of peptide self-assembly. Lim and coworkers have developed an infrared (IR)-responsive self-assembling peptide–carbon nanotube hybrid material that enables spatiotemporal control of bioactive ligand multivalency and subsequent human neural stem cell (hNSC) differentiation (Figure 2). These hybrid materials exquisitely integrate the non-covalently assembled peptide ligands and the thermo-responsive dendrimers to remotely control multivalency. In this organic–inorganic hybrid, carbon nanotubes (CNTs) were used as a photothermal trigger. By IR-induced photothermal conversion of ligand density, these peptide–CNT hybrid materials organized the integrins into nanoscale clusters and subsequently induced functional neuronal differentiation of hNSCs [86].

The enzymatic control of phosphorylation/dephosphorylation of the peptide can also regulate the formation of nanostructures. In the recent study by Shi and colleagues [87], enzymatic dephosphorylation of the amphiphilic peptide PP1 (VKVKVKVKV^D^PPTK_P_TEVKVKV), which contains a phosphorylated threonine, affected the folding of the peptide. As PP1 was dephosphorylated by phosphatase, the conformational equilibrium was shifted to the β-sheet conformation favoring the folded hairpin structure in the self-assembled fibril network. However, phosphorylated PP1 formed a gel with increased stiffness, resulting in broken fibril networks. This suggests that the use of enzymes can be a tool to control the self-assembly of peptides and affect the construction of nanostructure.

## 4. Application of Self-Assembling Peptide in Disease Treatment

The building blocks discussed above have been used to produce various types of structures based on material properties and environmental factors. Over the last few decades, the practical application of peptide-based self-assembled structures has been attempted widely. Table 2 summarizes nanostructures formed by particular self-assembling peptide blocks and their applications for specific biomedical uses.

### 4.1. Application in Cancer Treatment

As a conventional cancer treatment, chemotherapy is fairly successful, but the off-target side effects causing damage to healthy cells and unavoidable development of multi-drug resistance are problematic [111,112]. The response of self-assembling peptides to environmental conditions may offer a means to prevent the aforementioned issues, because the tumor environment has a lower pH and higher temperatures than normal tissues. Thus, self-assembling peptides are well suited for controlled release or targeting of anticancer drugs to tumor sites [113].

#### 4.1.1. Targeting

Chemotherapy in conventional cancer treatment is not only targeted to cancer cells, but also affects normal cells that are active in division and proliferation, such as bone marrow, hair, the mucous membrane of the gastrointestinal tract, and reproductive cells. To minimize the nonselective side effects, a specific peptide sequence or motif can be used in chemotherapy. Peptides designed as nanoparticles for targeting cancer cell surfaces or tumor vasculature in chemotherapy can be used to minimize systemic drug exposure and increase efficiency [114]. One of the cancer-targeting sequences, RGD, which binds to integrin, originates from a cell surface glycoprotein. RGD peptides can be linked to self-assembling peptides and increase the targeting effect of therapeutic drugs [115]. Furthermore, cyclic RGD increases binding affinity to integrins and is helpful in targeting drugs to cancer cells. Murphy et al. showed that cyclic RGDfK used to increase doxorubicin targeting suppressed growth of the primary tumor and prevented metastasis [97]. Another cancer-targeting peptide is the peptide Lyp-1, -CGNKRTRGC-, a nine-amino acid cyclic peptide that recognizes lymphatic metastatic tumors and exerts cytotoxic activity. LyP-1-conjugated PEG–PLGA nanoparticles (LyP-1-NPs) showed increased cellular uptake, by up to 4–8-fold in vitro and in vivo compared with PEG–PLGA nanoparticles without Lyp-1. LyP-1-NPs showed good targeting efficiency to cancer cells in vitro and to metastatic foci in vivo [92].

#### 4.1.2. Drug Delivery

By modulating the properties of self-assembling peptides, drug release rates can be efficiently controlled. Ketone-containing drugs linked to peptide amphiphiles can be sustainably released at physiological pH. For example, doxorubicin or paclitaxel containing ketone functionality allows covalent tethering between the drug and peptide amphiphile via addition of a hydrazino acid to a lysine ε-amine [98]. In a study from the Stupp group, the peptide amphiphile C16V2A2E2K(Hyd) and its modifications were successfully bound to the ketone-containing fluorescent compound, Prodan. The release rate was dependent on the packing density, the order of the hydrophobic peptide amphiphilic core, and the β-sheet character of the peptide. It can also be controlled by chemical properties (Log P, pKa, pI, presence of aromatic rings, and steric hindrance) and by the solvent release [116]. RADA16 was also used in hydrophobic drug delivery and for self-assembling hydrogels. The diffusion properties of pindolol, quinine, and timolol maleate, through RADA16, showed sustained, controlled, reproducible, and efficient drug release [101,102]. RADA16-X controls the release of hydrophobic antitumor drugs and effectively inhibits the growth of a breast cancer cell line [7]. In this case, hydrogels with a higher peptide concentration have a longer release half-life and could block tumor cell proliferation more effectively.

As drug delivery carriers, self-assembling peptides offer many advantages, such as high efficiency of drug loading, a low ratio of drug loss, and high stability that avoids body clearance [117]. For example, EAK-16II can stabilize ellipticine, a hydrophobic anticancer drug, and form microcrystal suspensions in aqueous solutions. In particular, it is an ionic-complementary peptide that does not cause an immune response when applied to animals [100]. Curcumin, which has anti-inflammatory and antitumorigenic properties, has low water solubility and bioavailability, and is therefore difficult to use for therapeutic purposes. Self-assembling peptide hydrogels, such as the MAX8 (VKVKVKVKV^D^PPTKVEVKVKV-NH2) peptide, can be an effective vehicle for the delivery of curcumin [93]. This peptide makes β-hairpin hydrogels for injectable agents to provide local curcumin delivery. The study with a medulloblastoma cell line confirmed that the encapsulation of curcumin with a hydrogel did not interfere with its drug activity. Peptide-based nanostructures made from polylactide (PLA) and VVVVVVKK (V6K2) are also used for the drug delivery of doxorubicin and paclitaxel [99]. The release of doxorubicin from PLA–V6K2 nanoparticles was slower than that from PLA–ethylene glycol nanoparticles. In addition, PLA–V6K2 nanoparticles showed a significantly increased cellular uptake rate with no induction of cytotoxicity in marrow stromal cells. However, it was more toxic to the 4T1 mouse breast carcinoma cell line than free doxorubicin. Moreover, PLA–V6K2 nanoparticles exhibited higher tumor toxicity and lower host toxicity in syngeneic breast cancer cells inoculated in mice, suggesting efficient drug delivery with selective toxicity.

### 4.2. Application in Regenerative Medicine

Regenerative medicine requires biocompatible scaffolds to increase cell engraftment and improve functionality, as well as to enhance cellular delivery processes. In recent years, engineering of nanostructures formed by self-assembling peptides can function as a scaffold in vivo.

#### 4.2.1. Self-Assembling Peptides for Hepatocyte Regeneration

The liver is the only regenerative visceral organ in mammals. Self-assembled peptide scaffolds can assist cell growth with increased implantation rate, resulting in fully differentiated hepatocytes. RADA16-I peptide was used for the 3D culture of Lig-8 liver progenitor cells. The functional differentiation of hepatocytes by RADA16 scaffold was confirmed by the successful induction of CYP1A1 and CYP1A2 after hepatocyte cluster formation [103]. In addition, strongly hydrophobic peptide amphiphiles, such as KLD12 (AcN-KLDLKKDLKLDL-CNH2) or KFE8 (FKFEFKFE), resulted in greater stiffness of scaffold and increased cell growth, with a better differentiation rate compared with RADA16. From these results, the stiffness of self-assembled peptide scaffolds was shown to be one of the important factors for hepatocyte differentiation. An in vivo experiment with another hepatocellular carcinoma cell line, HepG2, also showed that RADA 16-I had a positive effect on cell growth and cluster formation [104]. RADA16 and other peptides combined with RADA16, such as RADA16-GRGDS, showed that myofibroblast replacement and hepatocyte cell proliferation were enhanced in vivo by RADA16 with an extended motif [118]. This result suggests that self-assembled peptide structures binding to other ECM motifs may be effective for liver tissue regeneration, although the enhancement of liver tissue regeneration requires various other forms of combined motifs.

#### 4.2.2. Self-Assembling Peptides for Neuronal Regeneration

The regeneration or rehabilitation of neuronal tissue is difficult, owing to the characteristics of neuronal cells. Neuronal cells grow slowly, are difficult to differentiate, and have great networking complexity in vivo. Therefore, it is difficult to recover from ischemia or stroke damage in the brain, which manifest as neural function loss and cell death. Moreover, clinical conditions with chronic or idiopathic neuronal degeneration, such as Alzheimer’s disease, Parkinson’s disease, and prion disease, remain incurable [119].

Previous studies using RADA16-I and II as scaffolds in immature mouse cerebellum and rat hippocampus cells showed successful extensive neuron outgrowth. This growth was sustained for more than 4 weeks and resulted in the formation of an active synaptic connection [105]. The -IKVAV- penta-peptide combined with -Glu- and A4G3-alkyl residues is another example of using self-assembling peptides in neuronal cell regeneration, because the -IKVAV- peptide, a laminin epitope, was previously shown to promote neurite sprouting and growth. This peptide not only increased the fibril formation of self-assembled structures, but also improved cell growth, promoted neuronal cell differentiation, and inhibited astrocyte differentiation [120]. In addition, -SKPPGTSS, -PFSSTKT-, and RGD motifs combined with the RADA16-1 peptide increased the levels of nestin, β-tubulin, and other neuronal markers [106,107]. The self-assembling peptide nanofiber scaffold (SAPNS) composed of RADA16 resulted in reconnection of the injured spinal cord and facilitated axon regeneration and, eventually, locomotor functional recovery in animal models of spinal cord injury and acutely injured rat brain [121].

#### 4.2.3. Self-Assembling Peptides for Cartilage Regeneration

Traumatic injury or degenerative articular cartilage defects require repair, with the deposition of ECM on the bone or previously existing cartilage. For successful cartilage regeneration, the newly assembled ECM must be combined with the remaining cartilage to achieve stable elastic restoration. The self-assembling peptide hydrogel scaffold is useful as a template for chondrocyte proliferation and ECM accumulation. In an experiment using the self-assembling KLD-12 peptides, chondrocytes showed increased proliferation and greater accumulation of cartilage-specific ECM molecules, such as proteoglycans, in the hydrogel scaffold [69,109], indicating that a highly porous hydrogel can be applied as a good scaffold for cartilage regeneration. In another report about alternating polar and non-polar amino acid residues, the -FEFEFKFK- octarepeat peptide formed β-sheet-rich nanofiber scaffolds and improved chondrocyte differentiation and ECM accumulation efficiency compared with previously studied RADA16 or KLD-12 [110].

#### 4.2.4. Self-Assembling Peptides for Vascular Regeneration

Vascular regeneration aims to reshape blood vessels of various sizes and shapes, from microvascular to aortic [122]. Self-assembling peptides are also effective in vascular tissue regeneration and work as a scaffold to influence cell alignment, adhesion, and differentiation, and to promote better endothelization. In the study of Stupp et al. [89,90], peptide amphiphiles with a heparin-binding motif showed increased hierarchical structures and promoted rapid angiogenesis through heparin-binding growth factors involved in angiogenesis signaling. A Q11 (Ac-QQKFQFQFEQQ-Am) self-assembling peptide gel also promoted the proliferation of human umbilical vein endothelial cells and the expression of CD31 (PECAM), a vein endothelial cell marker, on the surface of these gels [91]. Both of these designed peptide structures helped the delivery and accumulation of angiogenic growth factors, such as VEGF and heparins, at the vessel regeneration site. RADA16 combined with the heparin binding domain motif sequence LRKKLGKA also increased VEGF delivery after myocardial infarction, and eventually improved cardiac function [94].

### 4.3. Other Applications

The BBB is the most challenging biological membrane encountered in drug delivery. Following the development of peptide science, researchers have tried to design self-assembling peptides to cross the BBB. The Tat (YGRKKRRQRRR) peptide found in a viral protein is known to penetrate the plasma membrane of cells, suggesting its function as a carrier to deliver drugs across the biological membrane. The Tat-polyethylene glycol-b-cholesterol (Tat-PEG-b-col) peptide, which forms micelles, allows antibiotics to migrate through the BBB [95]. Self-assembled polymersomes formed with lactoferrin-modified polyethylene glycol-poly (_D,L_-lactic-co-glycolic acid)(PEG–PLGA) successfully deliver humanin, a neuroprotective peptide, across the BBB in a rat model of Alzheimer’s disease [96].

Antimicrobial cationic peptides are used as peptide drugs to treat bacterial infection or prevent it. Antimicrobial peptides are amphipathic and destroy the cell membrane. For example, C16-KKK showed stronger antimicrobial activity than gentamicin in *E. coli* 25922 [123]. In another study, an antimicrobial cationic drug combined with RADA16 was shown to be released until 28 days after treatment [124]. Moreover, it also inhibited the growth of *Staphylococcus aureus*. Furthermore, cholesterol-conjugated Tat peptide (cholesterol–CG3R6TAT) formed nanoparticles that showed strong antimicrobial activity. It inhibited the growth of various types of Gram-positive bacteria, fungi, and yeast. These nanoparticles were able to cross the BBB and successfully treat *Staphylococcus aureus*-induced meningitis in rabbit [125].

The design of the self-assembling peptide scaffolds VEVK9 (Ac–VEVKVEVKV–CONH_2_), VEVK12 (Ac–VEVKVEVKVEVK–CONH_2_) and variants modified with RGD or a cell adhesion sequence resulted in a significant acceleration of fibroblast migration [88]. Periodontal ligament fibroblasts also significantly increased the production of Type I and Type II collagen upon culture with RADA16 combined with RGD and a laminin cell adhesion motif [108]. These results suggest that designed self-assembling peptides can regulate fibroblast cellular regeneration or reconstitution of the ECM. They can improve the skin or fibroblast tissue regeneration in scars or wound healing.

### 4.4. A New Paradigm of Nanostructure Formation with Reverse Self-Assembly

**Figure 3:**
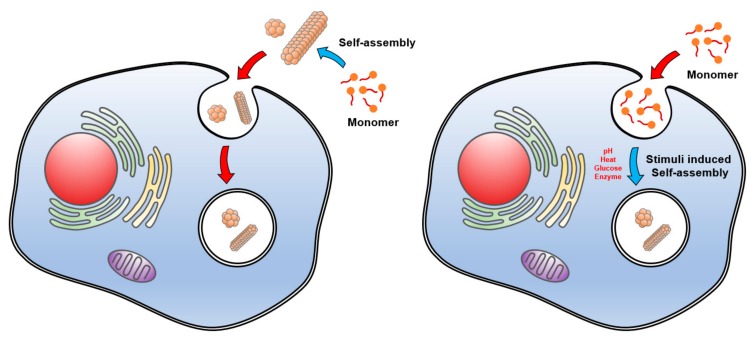
Concept of conventional forward (left) and novel reverse (right) self-assembly of peptides. Cellular environment and extrinsic stimuli such as endogenous changes of pH, glucose, enzyme activity, and heat drive in-cell self-assembly of peptides in the target sites.

Based on the understanding of self-assembling peptides, a strong interest in optimizing the self-assembly of these peptides has emerged. In recent years, a new strategy by which self-assembling peptide monomers assemble inside a living cell has been proposed (Figure 3). The term “reverse self-assembly” has been coined to describe the new strategy, because this process is distinct from the conventional approach in which self-assembling peptides first form nanostructures in vitro, and then peptide nanostructure is provided to the cells. In reverse self-assembly, self-assembling peptides (or prodrugs) are first administered in a monomer form at the pre-structured states, specifically concentrated at target sites, and spontaneously convert to self-assembled nanodrugs. For instance, when the FF peptide conjugated to tri-phenyl-phosphonium, the mitochondria targeting motif, is provided to cells, self-assembled peptide structures are exclusively accumulated in mitochondria [126].

Reverse self-assembly can be triggered by in situ stimuli, such as the induction of intracellular enzyme activity in a certain cell type or pH differences in a particular subcellular compartment. Enzyme-instructed self-assembly (EISA) is a useful strategy to accomplish intracellular reverse self-assembly. EISA is characterized by the self-assembly of monomers in cells or subcellular organelles, owing to endogenous enzymes targeted on-site. For instance, the hydrophilic precursor of hydrophobic FF peptide containing an esterase-cleavable bond provided to cancer cells is cleaved by intracellular esterase, resulting in formation of FF fibrils and gels within the cells, which induces cancer death [127]. Similarly, in-cell dephosphorylation of phosphorylated NDP1 (4-nitro-2,1,3-benzoxadiazole (NBD)-conjugated D-peptide) by intracellular alkaline phosphatase (AP) in some cancer cell lines, including HeLa cells, results in formation of non-diffusive nanofibrils [128]. Furthermore, self-assembled fibrils are formed in both pericellular space and endosomes, in which AP expresses abundantly. Compared with normal tissues, tumors exhibit a consequence of abnormal metabolism, such as increased temperature, decreased pH, induced expression of proteins and enzymes, and enhanced glycolysis. Because normal cells do not express AP as much as cancer cells do, reverse self-assembly of dephosphorylated NDP1 rarely occurs in normal cells, suggesting a feasibility of targeted drug delivery via in-cell reverse self-assembly. In addition, EISA is useful for controlled drug release in cells. Intracellular phosphatase activity facilitates in-cell formation of supramolecular hydrogels with Taxol-conjugated self-assembling peptide monomers, resulting in slow release of Taxol, a drug with low solubility, from the reverse assembled gels [129].

**Figure 4:**
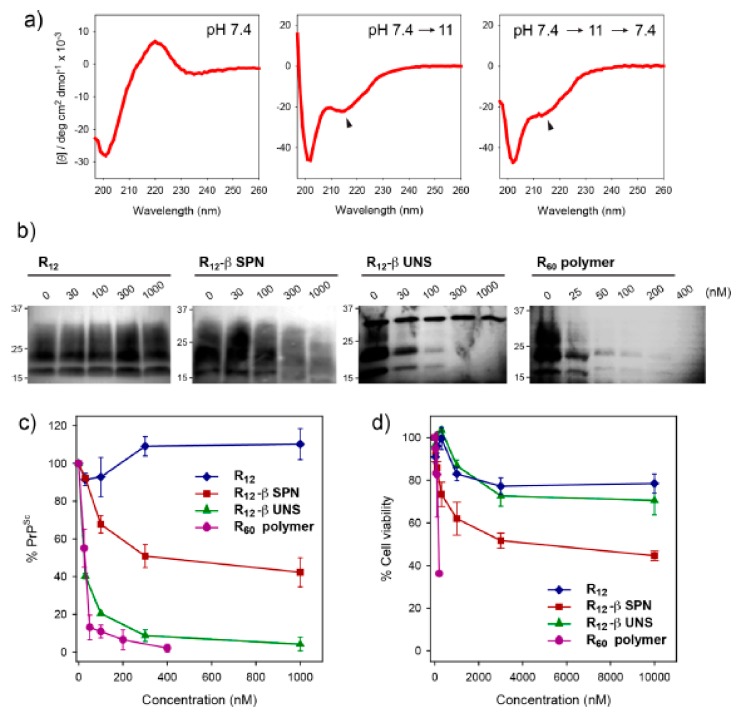
Improvement of anti-prion activity and cytotoxicity with reverse self-assembly of peptide blocks. (**a**) In vitro simulation of self-assembly of R_12_-FKFEFKFEFKFE peptides as pH changes. (**b**) Western blots of prions of which level decreased by reverse self-assembly of R_12_-FKFEFKFEFKFE peptides (R_12_-βUNS). (**c**) Quantitative presentation of anti-prion activity of forward and reverse self-assembled R_12_-FKFEFKFEFKFE peptides. (**d**) Cytotoxicity of forward and reverse self-assembled R_12_-FKFEFKFEFKFE peptides. Reprinted with permission from [76].

**Figure 5:**
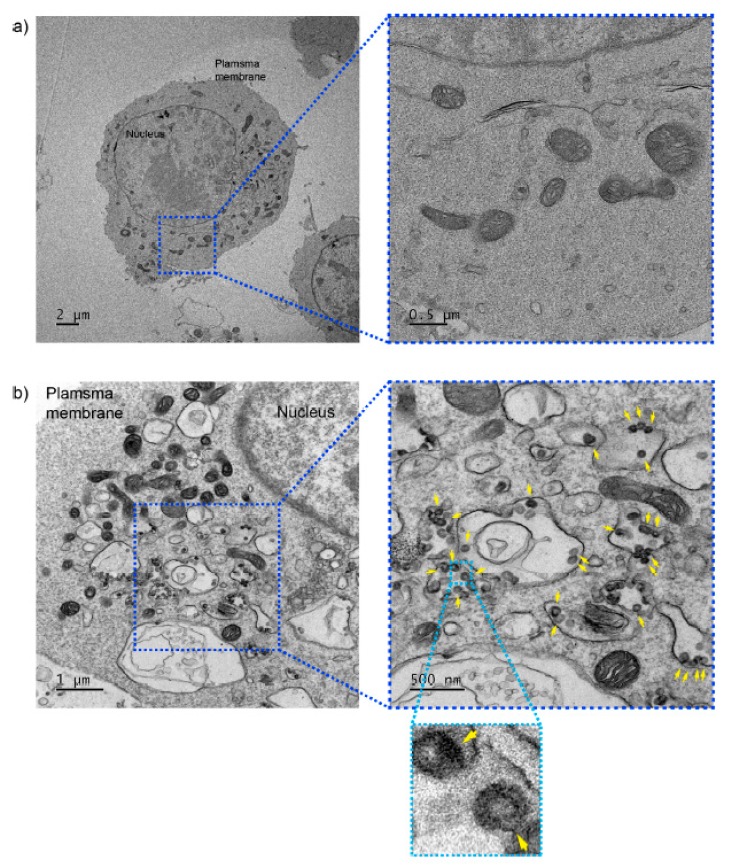
In-cell self-assembly of R_12_–FKFEFKFEFKFE peptide (R_12_-βUNS). Transmission electron micrographs of N2a neuroblastoma cells demonstrating reverse self-assembled nanostructure in the subcellular organelles. (**a**) Cells without incubation with the peptides. (**b**) Cells incubated with the peptides. Arrows indicate vesicle formed in the subcellular organelles. Reprinted with permission from [76].

Another strategy of reverse self-assembly is the employment of pH responsiveness of self-assembling peptides in the acidic subcellular compartments. Waqas and colleagues demonstrated in-cell self-assembly of a -FKFEFKFEFKFE β-sheet-forming peptide motif combined with an oligo-arginine R_12_ peptide, which resulted in stronger anti-prion effects with improved cytotoxicity (Figure 4) [76]. In-cell self-assembled R_12_-βUNS successfully decreased prions effectively to the level of anti-prion activity of the large oligo-arginine R_60_, which was much improved from that of R_12_-βSPN, the self-assembled nanostructure by “forward self-assembly”. Similarly, reverse self-assembled R_12_-βUNS improved cytotoxicity, exhibiting toxicity similar to that of the small oligo-arginine R_12_, but lower than that of forward self-assembled R_12_-βSPN. More interestingly, the R_12_-FKFEFKFEFKFE peptide monomers (R_12_-βUNS) provided to the cells formed nanostructures in the subcellular organelles (Figure 5). These result suggest that reverse-assembled peptide nanostructure can be used as an efficient therapeutic tool to circumvent the hurdles found in the application of forward-assembled peptide nanostructure in disease treatment.

## 5. Conclusions

In this review, we have summarized the characteristics, structures, and regulatory factors of self-assembled peptides and their self-assembled nanostructures. In addition, the application of self-assembling peptides to various diseases has been summarized in the context of cancer, regenerative medicine, and other diseases. Although self-assembling peptide drugs are rarely used clinically, drug delivery and motif design have been studied for several drugs. The biocompatibility and biodegradability of peptide materials are advantageous for targeted drug development and in vivo scaffold development. Thus, further developments in this field are expected in the future. Finally, the limitations of conventional forward self-assembled peptide structures associated with charge or size can be overcome through in-cell reverse self-assembly of peptides.

## Figures and Tables

**Table 1:** Peptide building blocks that self-assemble.

Peptide Building Blocks	Characteristics	References
Dipeptides	Simple phenylalanine dipeptides with or without N-terminal modifications, such as N-fluorenylmethoxycarbonyl (Fmoc) and naphthyl	[16,17,18,19]
Surfactant-like peptides	Amphiphilic structure with both hydrophilic and hydrophobic amino acids included in the peptide head and tail	[20,21,22]
	Repeated sequence of hydrophobic amino acids	
Peptide amphiphiles with an alkyl group	An alkyl tail linked to the N- or C-terminus	[23,24]
	A hydrophilic functional region	
	Form a stable β-sheet, providing hydrogen bonds for self-assembly	
	Glycine linker residues support flexibility	
Bolaamphiphilic peptides	Two hydrophilic heads connected by a hydrophobic region that is generally composed of alkyls	[25,26,27,28,29,30]
Ionic-complementary self-assembling peptides	A hydrophobic tail promotes self-assembly in water	[31,32,33,34]
	A hydrophilic tail with charged amino acids residues forms an ionic bond	
	Classified by the number of repeated ion charges: Type I has a charge pattern of “+-+-+-”, Type II has “++--++--“, Type III has “+++---+++”, and Type IV has “++++----“.	
Cyclic peptides	Even number of alternating D and L amino acids stacked by hydrogen bonding	[35,36,37,38,39]
	Other types of cyclic peptides are characterized by amphiphilic characteristics, i.e., one side of the cycle is hydrophilic, whereas the other side contains hydrophobic and/or aggregation-prone amino acids	

**Table 2:** List of self-assembling peptide sequences and resultant nanostructures used for disease treatment.

Structure	Sequence	Applications	Reference
Nanofibers	VEVK9 (VEVKVEVKV) and VEVK12 (VEVKVEVKVEVK)/combined with RGD	Increase fibroblast migration	[88]
	V3A3E3 (VVVAAAEEE)	Stem cell culture and differentiation	[23,46]
Nanotubes	Heparin-binding peptide amphiphile (HBPA)	Hierarchical structure	[89,90]
	Q11 (QQKFQFQFEQQ)	Endothelial cell proliferation	[91]
Nano particle, vesicle, micelle, suspension	Lyp-1 (CGNKRTRGC)	Increase drug cellular uptake	[92]
	MAX8 (VKVKVKVKV^D^PPTKVEVKVKV)	Drug delivery	[93]
	RADA16 with LRKKLGKA	Vascular endothelial growth factor (VEGF) delivery to the myocardium	[94]
	Tat/Tat combined with PEG/Cholesterol	Cross blood brain barrier (BBB)drug delivery	[95,96]
	cRGDfK	Drug targeting	[97]
	C16V2A2E2K(Hyd)	Drug stabilization	[98]
	V6K2(VVVVVVKK) combined with PLA	Drug delivery	[99]
	EAK16II (AEAEAKAKAEAEAKAK)	Drug stabilization	[100]
Hydrogel	RADA16I (RADARADARADARADA)	Controlled drug release	[101,102]
	RADA16I (RADARADARADARADA)	Hepatocyte regeneration	[103,104]
	RADA16 II (RARADADARARADADA)	Neuron regeneration	[105]
	RADA16-I combined with RGD motif	Neuron regeneration	[106,107]
	RADA16-I combined with RGD motif	Ligament regeneration	[108]
	KLD12 (KFDLKKDLKLDL)	Hepatocyte regeneration	[103]
	KLD12 (KFDLKKDLKLDL)	Chondrocyte regeneration	[69,109]
	KFE8 (FKFEFKFF)	Hepatocyte regeneration	[103]
	FEFEFKFK octarepeat	Extracellular matrix (ECM) accumulation	[110]
